# Detection of *Salmonella enterica* subsp. *enterica* via Quenching of Unincorporated Amplification Signal Reporters in Loop-Mediated Isothermal Amplification

**DOI:** 10.1155/2022/4567817

**Published:** 2022-12-30

**Authors:** Ma. Hanna S. C. Halcon, Joy Ann P. Santos, Nacita B. Lantican

**Affiliations:** ^1^Graduate School, University of the Philippines, Los Banos College, Los Banos, Laguna 4030, Philippines; ^2^Research and Biotechnology Division, Manila HealthTek Inc., 109 Gil Fernando Ave, Marikina 1800, Philippines; ^3^Biological Research and Services Laboratory, Natural Sciences Research Institute, University of the Philippines Diliman, Quezon, Metro Manila 1104, Philippines; ^4^Microbiology Division, Institute of Biological Sciences, University of the Philippines, Los Banos College, Los Banos, Laguna 4031, Philippines

## Abstract

*Salmonella enterica* is a major cause of diarrheal diseases in developing countries where timely surveillance and proper clinical management are inadequate. In this study, a rapid and cheap method of detecting *S. enterica* DNA was developed by employing the Quenching of Unincorporated Amplification Signal Reporters in Loop-Mediated Isothermal Amplification (QUASR LAMP) platform. QUASR LAMP provides a closed-tube, target-specific endpoint detection of pathogens, wherein results can be analyzed visually through an LED transilluminator and verified through agarose gel electrophoresis. Based on the chromosomal SopD gene, primers and probes were first designed, then screened. The assay was subsequently optimized so that the presence of *S. enterica* is determined by incubating the extracted DNA at 65°C for only 60 minutes. The assay was repeatable and can be performed by simply using a thermal cycler or even a dry bath incubator. *S. enterica* positives appear bright yellow green when viewed through a yellow filter excited with blue LED. The developed assay had an *in silico* and *in vitro* specificity towards *Salmonella enterica* subsp. *enterica* serovars with a limit of detection of 10^4^ copies per microliter. The *Salmonella* QUASR LAMP assay has the potential for food and environmental applications. Chiefly, as an alternative to traditional microbiology and PCR, this QUASR LAMP assay can be used for point-of-care salmonellosis testing of clinical specimens in low-resource settings.

## 1. Introduction

*Salmonella enterica* is one of the most common causes of food-borne illnesses in developed countries and the major cause of diarrheal diseases in developing countries [1]. Aside from being the infectious agent of typhoid fever, *S. enterica* is also known to cause bacterial food poisoning. Nontyphoidal *Salmonella* (NTS) causes self-limiting diarrhea in an immunocompetent host. For an immunocompromised host, it can develop into a systemic disease. NTS infections are a major global threat, afflicting an estimated ninety-three million people annually worldwide [2]. Salmonellosis in humans is generally contracted through the consumption of contaminated foods of animal origin (i.e., eggs, meat, poultry, and milk), although other foods, including green vegetables contaminated by manure, have been implicated in its transmission [3]. Therefore, surveillance systems on food-borne diseases such as salmonellosis are necessary to detect and respond to enteric infections in their early stages and thus prevent them from further spreading.

*S. enterica* possesses many virulence strategies employed to interact with the host defense mechanisms. The SopD effector protein, encoded by the chromosomal SopD gene, presumably increases inflammation and fluid secretion during gastroenteritis by directly promoting *Salmonella* invasion [1]. SopD also plays a role during desiccation survival which makes it survive several weeks in a dry environment and several months in water [4, 5]. Unfortunately, current diagnostics for *Salmonella* infections, including invasive NTS, are inadequate to guide timely surveillance and proper clinical management [6, 7].

Aside from the standard culture methods, nucleic acid amplification tests (NAATs) can be used to detect DNA in biological samples to detect contamination and diagnose pathogenic infection from *S. enterica* [8]. NAATs are used as a staple molecular diagnostic for infectious diseases [9–11]. Common NAATs include conventional polymerase chain reaction (PCR) and quantitative PCR (qPCR). qPCR provides great sensitivity and precision [12]. However, it requires a well-equipped laboratory and, thus, poses a major challenge for point-of-care applications. Moreover, qPCR requires highly purified extracted nucleic acid, cold-chain reagents, and nonportable instrumentation that usually demands high electrical power [10].

**Figure 1:**
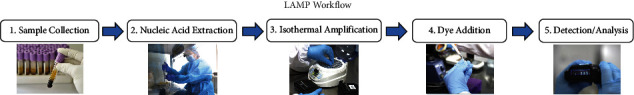
Typical loop-mediated isothermal amplification (LAMP) workflow in clinical diagnosis setting. (1) A clinical sample (e.g., blood, serum, stool, nasal swab) from the patient is first collected and then subjected to (2) nucleic acid extraction, where the DNA or RNA from the sample is isolated, purified, and concentrated. (3) The extracted nucleic acid is then added to the LAMP reaction mixture and incubated isothermally for 60 minutes. (4) After amplification, the reaction tube is opened, and an intercalating dye (SYBR green dye) is put into the mixture. (5) Analysis can be done using the naked eye or by viewing through a blue LED light. Using the naked eye, positives appear yellow-green, and negatives appear orange. Through a blue LED light, positives appear as bright fluorescent yellow green, and negatives appear as dull and colorless. Agarose gel electrophoresis can also be performed to confirm the amplification of target products. Ladder-like bands are characteristic of LAMP products.

**Figure 2:**
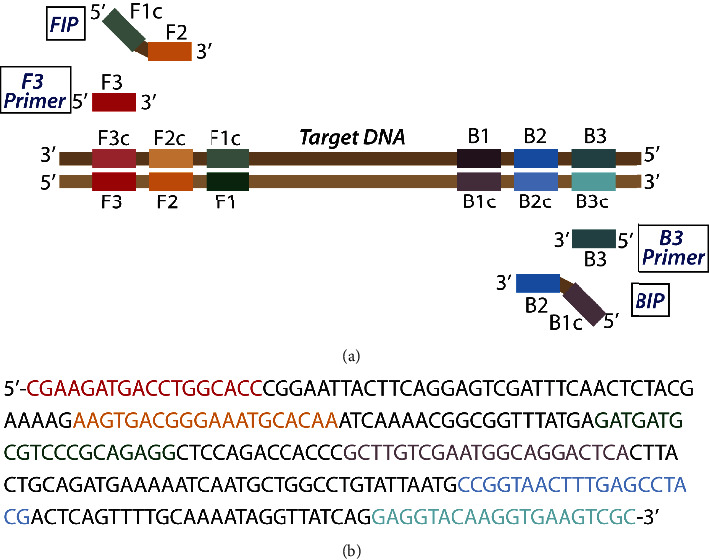
(a) LAMP primer design. Six distinct regions are designated on the target DNA, labeled F3, F2, F1, B1c, B2c, and B3 from the 5′ end. The F1c sequence is complementary to the F1 sequence. Two inner primers (FIP and BIP) and two outer primers (F3 and B3) are used in the LAMP method. FIP (BIP) is a hybrid primer consisting of the F1c (B1c) sequence and the F2 (B2) sequence. (b) LAMP primer mapping. The portion of the SopD gene that is targeted by the developed *Salmonella enterica* QUASR LAMP assay is shown here. The colored nitrogenous bases show the corresponding LAMP primer in Figure 2(a).

In contrast, loop-mediated isothermal amplification (LAMP), an isothermal nucleic acid amplification technique, offers a useful alternative to PCR for low-cost diagnostics for infectious diseases (Figure 1) [13]. This technique utilizes primer-based amplification of DNA and RNA targets (Figure 2(a)) [14]. This type of NAAT is robust and sensitive and can work with minimal or no sample treatment. However, the available detection techniques used in LAMP are not easily amenable to multiplexing to distinguish multiple targets in a single reaction [15]. LAMP results are typically analyzed by running the product on a gel [16] or by adding a dye post-reaction [17, 18], which requires opening the tube after amplification and presents a risk for amplicon contamination (Figure 1). Thus, most of LAMP's detection mechanisms are nonspecific and prone to false positives.

**Figure 3:**
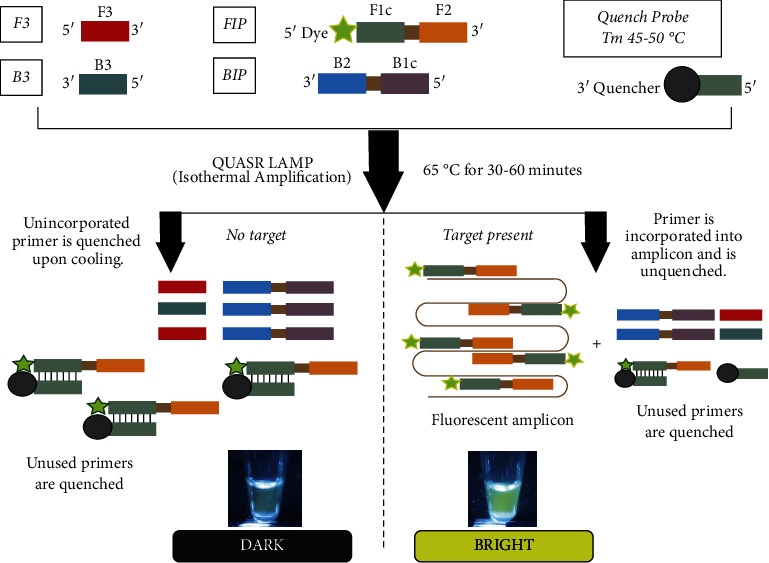
Principle of quenching of unincorporated amplification signal reporters (QUASR) detection in LAMP. One of the loop primers (LF or LB) or inner primers (FIP or BIP) is labeled with a dye. The reaction mixture also contains a short probe, labeled with a dark quencher at the 3′ end and complementary to 7–13 bases at the 5′ end of the dye-labeled primer. The quench probe is present in slight excess relative to the labeled primer and has Tm > 10°C below the temperature of the LAMP reaction, such that it remains dissociated during the amplification. After incubation, the reaction is cooled to ambient temperature, resulting in the dark quenching of fluorescent primers (negative reactions) or highly fluorescent amplicons (positive reactions) [16]. Adapted from More with Less: Novel Approaches to LAMP Assay Design for Better Performance with Fewer Resources by R. Meagher, 2019.

**Figure 4:**
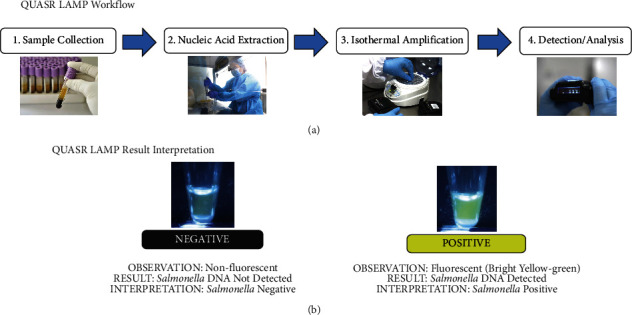
(a) Typical QUASR LAMP workflow in clinical diagnosis setting. The QUASR LAMP workflow is like the LAMP workflow (Figure 1) without the dye addition step. Not opening the tube after amplification makes QUASR LAMP less prone to contamination and false positives. (b) QUASR LAMP interpretation of results. Analysis can be done by viewing the reaction tubes through a blue LED light with a yellow filter. Positives appear bright fluorescent yellow green, and negatives appear nonfluorescent and dull.

A novel approach for the endpoint determination of LAMP is based upon the quenching of unincorporated amplification signal reporters (QUASR) [19]. In QUASR, one of the primers in the LAMP reaction is labeled with a dye (Figure 3). Compared to traditional LAMP, wherein detection is nonspecific through the addition of a dye, QUASR LAMP offers sequence-specific detection because the fluorescent dye is already appended to the primer. Additionally, the reaction mixture contains a short probe, labeled with a dark quencher at the 3′ end, which is complementary to 7–13 bases at the 5′ end of the dye-labeled primer. The quench probe is present in slight excess relative to the labeled primer and has Tm > 10°C below the temperature of the LAMP reaction so that it remains dissociated during the amplification. After incubation, the reaction is cooled to ambient temperature, resulting in dark quenching of fluorescent primers (i.e., negative reactions), or highly fluorescent amplicons (i.e., positive reactions) [19]. By combining multiple QUASR LAMP primer sets specific for different targets, spectrally multiplexed detection can be achieved [19]. QUASR is reported to provide a closed-tube, target-specific endpoint detection (Figure 4). It has large discrimination between positive and negative samples and requires minimal instrumentation [19–21]. Therefore, QUASR can be suitable for deployable LAMP detection of *Salmonella enterica*. This study, therefore, aimed to demonstrate the feasibility of the QUASR LAMP assay in detecting *Salmonella enterica* in bacterial samples.

## 2. Materials and Methods

### 2.1. LAMP Primer Design

The SopD gene was targeted for detecting general *Salmonella enterica* subsp. *enterica*. Available *Salmonella enterica* subsp. *enterica SopD* gene sequences were obtained from the GenBank database. Primer Explorer v.5 software (Fujitsu Ltd. 2015) was used with default parameters to scan for suitable LAMP primer sets for the SopD gene (GenBank NZ_CP009102.1). Four types of LAMP primers were designed using Primer Explorer v.5 (Fujitsu Ltd, 2015) based on the following six distinct regions of the target gene, designated in Figure 2(b) on the right from the 5′-end as F3, F2, F1, B1, B2, and B3 (Table 1). The forward inner primer (FIP) consists of the F2 sequence (at its 3′ end) that is complementary to the F2c region and the same sequence as the F1c region at its 5′ end [16].

### 2.2. LAMP Primer Screening and Preliminary Assay

Before proceeding to QUASR LAMP optimization, the SopD primers were initially screened by LAMP. The assays were performed using clear 0.2 mL PCR tubes with flat caps with 5 *μ*L sample DNA as template in a total volume of 25 *μ*L containing 2.5 *μ*L 10 *μ*M Thermopol Buffer (New England BioLabs, Inc., Ipswich, MA, USA), 1.0 *μ*L 100 *μ*M MgSO_4_, 3.5 *μ*L 10 *μ*M dNTP (Promega, WI, USA), 3.0 *μ*L 5 M Betaine, 2.0 *μ*L 8 *μ*M Bst Polymerase (New England BioLabs, Inc., Ipswich, MA, USA), 2.0 *μ*L 10 *μ*M FIP and BIP primers (Macrogen, South Korea), 1.0 *μ*L 5 *μ*M F3 and B3 primers (Macrogen, South Korea), and 2.0 *μ*L nuclease-free water.

Reaction tubes were incubated at 60°C for 50 minutes, then stopped at 80°C for 2 minutes. LAMP results were analyzed visually using the naked eye and a blue-light LED illuminator. Results were also verified with agarose gel electrophoresis data showing the characteristic ladder-like patterns.

### 2.3. Fluorophore-Labeled Primer and Quencher Probe Design

To design the fluorophore-labeled primers for the QUASR LAMP reaction, the 5′ end of the FIP primers was appended with a fluorophore (Table 2). The FAM reporter dye (green fluorophore) was used for the SopD FIP. A short quencher probe complementary to the 5′ end of the labeled primer (FIPc) was manually designed. The quencher probes were modified at the 3′ ends with a dark quencher (e.g., Black Hole quencher [BHQ]). The quencher used for SopD FIP is BHQ-1. The designed quencher probes were analyzed such that they were not likely to form stable hairpins. Dye-labeled primers and quenching probes were ordered from Macrogen (Seoul, Korea).

### 2.4. Reference Strains

The nontyphoidal *Salmonella enterica* subsp. *enterica* sv. Typhimurium derived from ATCC 13311 (KWIK-STIK™ Plus Catalog No. 0421X, Microbiologics, Inc., MN, USA) was purchased from Fil-Anaserve (Quezon City, Philippines) and reconstituted according to the manufacturer's instructions. Tryptic Soy Broth (BD Diagnostics System Difco™ USA) samples of the typhoidal *Salmonella enterica* subsp. *enterica* serovars Paratyphi A (ATCC 9150), Paratyphi B (ATCC 8759), and Typhi (BIOTECH 1768) were obtained from the Natural Science Research Institute (University of the Philippines Diliman, Quezon City, Philippines). Pure bacterial cultures of *Escherichia coli* (BIOTECH 1634), *Staphylococcus aureus* (BIOTECH 1582), *Staphylococcus epidermidis* (BIOTECH 10098), *Pseudomonas aeruginosa* (BIOTECH 1335), *Klebsiella pneumoniae* (BIOTECH 1754), *Enterococcus faecalis* (BIOTECH 10348), and *Listeria monocytogenes* (BIOTECH 1958) were purchased from the Philippine National Collection of Microorganisms (National Institute of Molecular Biology and Biotechnology, UP Los Banos) and served as negative controls in subsequent *in vitro* specificity assays (Table 3).

### 2.5. DNA Isolation

Genomic DNA was extracted from bacterial cultures that were grown in Tryptic Soy Broth at 37°C for 24 h using the GenAmplifyTM DNA Purification Kit (Manila HealthTek, Inc., Marikina, Philippines) according to the manufacturer's instructions.

### 2.6. QUASR-LAMP Assay Optimization

After confirming that the primers designed were useable and specific to the target organism, the QUASR LAMP assay was optimized. The designed fluorophore-labeled primers and quencher probes were incorporated into the reaction mixture. Optimization was done to obtain the result with the brightest fluorescence in the positive control (PC) and the least background fluorescence in the no template control (NTC). All QUASR LAMP assays were done with 12.5 *μ*L reaction volumes in clear 0.2 mL PCR tubes with flat caps.  Figure 4(b) summarizes the QUASR LAMP assay interpretation for the target. *S. enterica* positives appear bright yellow green. All results were verified with gel electrophoresis data wherein positives show the characteristic ladder-like patterns.

The initial QUASR-LAMP formula used was based on a previously established functional formula for detecting other organisms. The assays were performed with 2.5 *μ*L sample DNA as a template in a total volume of 12.5 *μ*L containing 1.25 *μ*L 10 *μ*M Thermopol Buffer (New England BioLabs, Inc., Ipswich, MA, USA), 1.75 *μ*L 50 *μ*M MgSO_4_, 1.75 *μ*L 10 *μ*M dNTP (Promega, WI, USA), 1 *μ*L 5 M Betaine, 1.25 *μ*L 8 *μ*M Bst Polymerase (New England BioLabs, Inc., Ipswich, MA, USA), 0.5 *μ*L 40 *μ*M FIP and BIP primers (Macrogen, South Korea), 0.4 *μ*L 10 *μ*M F3 and B3 primers (Macrogen, South Korea), 0.5 *μ*L 60 *μ*M quencher probe (Macrogen, South Korea) and 0.7 *μ*L nuclease-free water. From this formula, the optimal incubation temperature was determined by performing gradient QUASR LAMP in a T100 Thermal Cycler (Bio-Rad Laboratories, Inc., CA, USA). The following protocol was used for gradient QUASR LAMP assays: amplification at 60–65°C for 50 min, stop reaction and Bst polymerase inactivation at 80°C for 2 min, then cool down at 4°C for a minimum of 5 min.

To determine the optimal amplification time for the reaction, different incubation periods (30, 40, 50, and 60 min) were evaluated. For the detection of *S. enterica* using the SopD primer set, the following was the optimized protocol which was used for subsequent tests: amplification at 65°C for 60 min, stop reaction and Bst polymerase inactivation at 80°C for 2 min, then cool down at 4°C for a minimum of 5 min. Reactions were incubated using a T100 Thermal Cycler (Bio-Rad Laboratories, Inc., CA, USA).

### 2.7. Repeatability

Repeatability expresses the precision under the same operating conditions over a short interval of time [23]. Three different assays were done by one operator over three days using three replicates of the no template control (NTC), the 10^6^ cp/*μ*L positive control (PC), and the *S. enterica* sv. Typhi DNA sample. Repeatability assays were performed using two setups, one using a T100 Thermal Cycler (Bio-Rad Laboratories, Inc., CA, USA) and another using a lab-in-a-mug dry bath incubator (Manila HealthTek, Inc., Philippines).

### 2.8. Analytical Characteristics

To confirm that the acquired SopD primer set would indeed amplify the target organism of interest, the outermost primers (F3 and B3) were subjected to *in silico* amplification. *In silico* PCR was performed using a freely available molecular biology resource for experiments against prokaryotic genomes (http://insilico.ehu.es/). The F3 and B3 primer sequences were input, and *in silico* PCR amplification was done against different bacterial genomes. The optimized QUASR LAMP assay was also tested *in vitro* against the positive and negative control panels listed in Table 3. Furthermore, the analytical sensitivity was determined based on three replicates of ten sample concentrations (10^9^, 10^8^, 10^7^, 10^6^, 10^5^, 10^4^, 10^3^, 10^2^, 10^1^, and 10^0^ copies of DNA per microliter (cp/*μ*L)) of an *S. enterica* sv. Typhi DNA sample.

## 3. Results and Discussion

### 3.1. QUASR-LAMP Assay Optimization

The key factors for LAMP primer design are melting temperature (Tm), stability at the 3′ and 5′ ends of each primer (ΔG), % guanine and cytosine (GC) content (optimal GC content range between 40% and 60%), and secondary structures. Moreover, since multiple primers anneal in different target regions at the same time, it is essential to consider the distance between primers and primer secondary structures such as self-dimers and cross dimers [22].

In designing the LAMP primers for this study, the Tm for each region was intended to be about 65°C (64–66°C) for F1c and B1c, and about 60°C (59–61°C) for F2, B2, F3, and B3. The 3′ ends of F2/B2 and F3/B3, and the 5′ end of F1c/B1c were designed so that the free energy (ΔG) is –4 kcal/mol or less. Primers were also designed so that their GC content is between 40% and 65%. To prevent the formation of primer dimers, it was ensured that the 3′ ends were not complementary. The distance from the end of F2 to the end of B2 (the region amplified by the LAMP method) is between 120 and 160 bases; the distance from the 5′ end of F2 to the 5′ end of F1 (the portion that forms the loop) is between 40 and 60 bases; and the distance between F2 and F3 is between 0 and 60 bases (A Guide to LAMP primer designing (PrimerExplorer V4). Eiken Chemical Co., Ltd.). Considering these key factors, the SopD primer set (Table 1) passed the initial LAMP screening and was used for further QUASR LAMP optimization.

LAMP is based on auto-cyclingstrand-displacement DNA synthesis carried out by Bst DNA polymerase large fragment under isothermal conditions [24]. Additionally, Bst DNA polymerase, an enzyme derived from *Geobacillus stearothermophilus*, has an optimal temperature ranging from 60°C to 65°C for general strand-displacement reactions and a deactivation temperature of 80°C [25]. As in PCR, factors such as incubation temperature and time are crucial for the *in vitro* amplification of nucleic acid targets in QUASR LAMP. The optimal incubation temperature for the assay was determined by performing gradient QUASR LAMP in a T100 Thermal Cycler using six incubation temperatures: 60°C, 61°C, 62°C, 63°C, 64°C, and 65°C.

**Figure 5:**
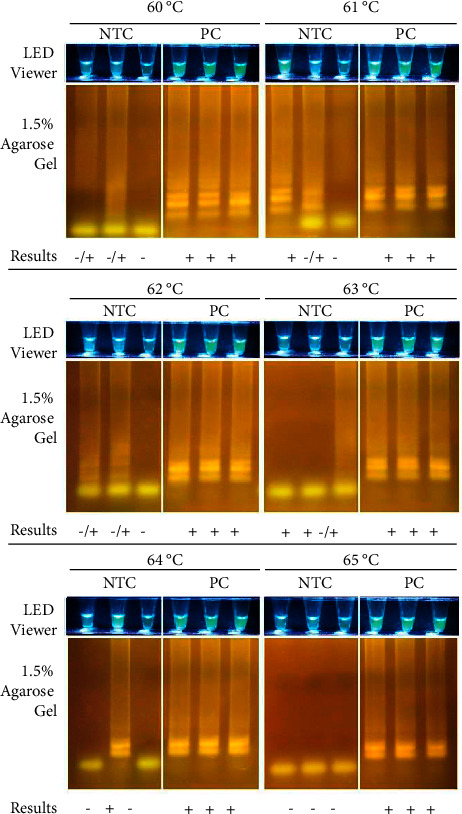
Optimization of assay incubation temperature. Sample (2.5 *μ*L) was added to 10 *μ*L reagent mix then incubated at 60–65°C for 60 minutes, 80°C for 2 minutes, and cooled to 4°C for 10 minutes using a T100 Thermal Cycler (S/N 621BR14432). Samples used were the No Template Control (NTC) and the Positive Control (PC; 10^6^ copies/*μ*L). Results were viewed using the EasyView™ LED Transilluminator (Manila HealthTek, Inc., Marikina, Philippines) which has a blue LED light and a yellow plastic gel filter, and through agarose gel electrophoresis (1.5% agarose, 135 V for 25 minutes). *Salmonella enterica* subsp. *enterica* positives appear bright yellow green. All images were taken using a Canon EOS 750D camera. Interpretation of results are as follows: (+)–Positive using the LED transilluminator and positive in 1.5% AGE. (−)–Negative using the LED transilluminator and negative in 1.5% AGE. (−/+)–Negative using the LED transilluminator but positive in 1.5% AGE.

Though the desired results were seen in the 60°C, 62°C, and 63°C setups (Figure 5), wherein negative reaction tubes (NTCs) appear dull and nonfluorescent while positive reaction tubes (PCs) appear bright fluorescent green, agarose gel verification data implies otherwise. In these temperatures, some of the NTC tubes showed ladder-like bands in 1.5% agarose gel which is characteristic of positive reactions. Moreover, at the 61°C and 64°C temperature points, one out of three NTC tubes showed a positive reaction in both the LED transilluminator and 1.5% AGE data. Therefore, the optimal temperature for the assay incubation is 65°C because NTC and PC reaction tubes yielded the expected results as seen in both the LED transilluminator and 1.5% AGE data (Figure 5).

**Figure 6:**
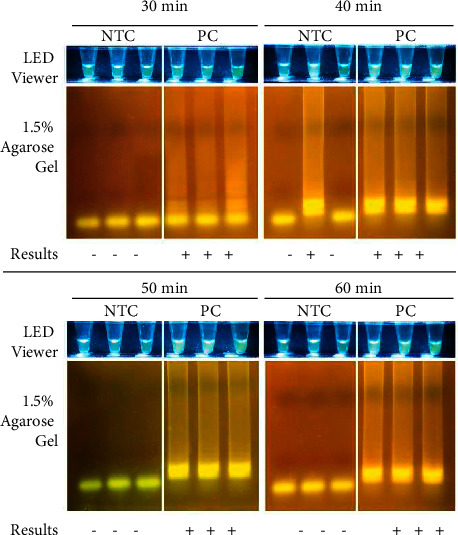
Optimization of assay incubation time. Sample (2.5 *μ*L) was added to 10 *μ*L reagent mix then incubated at 65°C for 30–60 minutes, 80°C for 2 minutes, and cooled to 4°C for 10 minutes using a T100 Thermal Cycler (S/N 621BR14432; Bio-Rad Laboratories, Inc., CA, USA). Samples used were the No Template Control (NTC) and the Positive Control (PC; 10^6^ copies/*μ*L). Samples were run in triplicate. Results were viewed using the EasyView™ LED Transilluminator (Manila HealthTek, Inc., Marikina, Philippines) which has a blue LED light and a yellow plastic gel filter, and through agarose gel electrophoresis (1.5% agarose, 135 V for 25 minutes). *Salmonella enterica* subsp. *enterica* positives appear bright yellow-green. All images were taken using a Canon EOS 750D camera. Interpretation of results are as follows: (+)–Positive using the LED transilluminator and positive in 1.5% AGE. (−)–Negative using the LED transilluminator and negative in 1.5% AGE. (−/+)–Negative using the LED transilluminator but positive in 1.5% AGE.

The optimal duration of the assay incubation was also determined by running the assay for 30, 40, 50, and 60 minutes (Figure 6). The 30-minute incubation time did not produce good contrast between NTC and PC reaction tubes. One NTC tube from the 40-min incubation setup showed a false-positive reaction. At the 50- and 60-min incubation points, the NTC and PC reaction tubes yielded the expected results, as seen in both the LED transilluminator and 1.5% AGE data. Thus, the optimal incubation time ranges from 50 to 60 minutes. The 60-min incubation period was selected for subsequent experiments since low input or inhibitor-containing samples may require extended incubation times to visualize detection (New England Biolabs, Inc., https://www.neb.sg).

From this point, all *Salmonella* QUASR LAMP assays were performed by incubating the reaction tubes at 65°C for 60 minutes, followed by 80°C for 2 minutes, then 4°C for 10 minutes. The Bst polymerase is generally deactivated at 80°C. Furthermore, cooling down at 4°C provides a chance for unused fluorescent primers to be quenched and prevents false positive detection.

### 3.2. Repeatability

Repeatability expresses the precision under the same operating conditions over a short interval of time [23]. To confirm if the assay can be performed using a simple dry bath incubator, repeatability assays were performed using two setups, one using a conventional thermal cycler and the other using a dry bath incubator.

**Figure 7:**
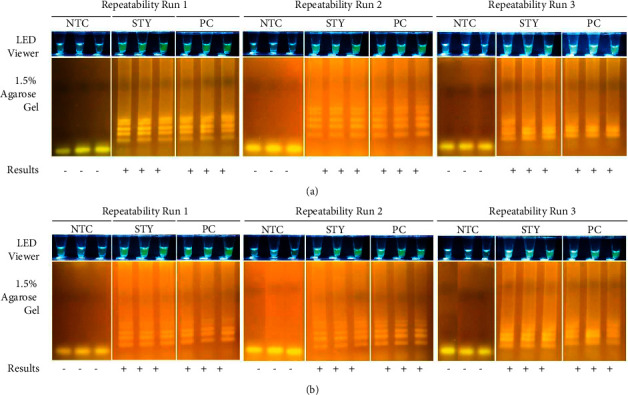
Assay repeatability. Three different QUASR LAMP assays were done by the same operator over three days using samples in triplicate. Sample (2.5 *μ*L) was added to 10 *μ*L reagent mix then incubated at 65°C for 60 minutes, 80°C for 2 minutes, and cooled to 4°C for 10 minutes using two machines: (a) T100 Thermal Cycler (S/N 621BR14432; Bio-Rad Laboratories, Inc., CA, USA) and (b) Lab-in-a-Mug 2.0 Dry Bath Incubator (Manila HealthTek, Inc., Marikina, Philippines). Samples used were the No Template Control (NTC), *Salmonella enterica* subsp. *enterica* serovar Typhi DNA (STY), and Positive Control (PC; 10^6^ copies/*μ*L). Results were viewed using the EasyView™ LED Transilluminator (Manila HealthTek, Inc., Marikina, Philippines) which has a blue LED light and yellow plastic gel filter, and through agarose gel electrophoresis (1.5% agarose, 135 V for 25 minutes). *Salmonella enterica* subsp. *enterica* positives appear bright yellow green. All images were taken using a Canon EOS 750D camera. Interpretation of results are as follows: (+)–Positive using the LED transilluminator and positive in 1.5% AGE; (−) – Negative using the LED transilluminator and negative in 1.5% AGE.

As seen in Figure 7, the *Salmonella* QUASR LAMP assay was repeatable for both setups.

Consistently for all replicates over three runs, the NTC reaction tubes did not show amplification and the PC reaction tubes showed positive amplification as seen in both the LED transilluminator and 1.5% AGE data for the two setups. Thus, there was an agreement between replicates within and between assay runs by the same operator over a short period of time [23]. This also underscores the simplicity of QUASR LAMP machinery, wherein it can be performed by laboratories with either a T100 Thermal Cycler or a dry bath incubator. As compared to the machine and power requirements of a real-time PCR, the testing laboratory in this scenario can thus cut down on expenses, especially in locations where stable electricity and budgetary allocations are limited.

### 3.3. Analytical Characteristics

To test the ability of the assay to exclusively identify the intended target, the analytical specificity was tested *in silico* and *in vitro*. The results of *in silico* PCR amplification using the online tool (http://insilico.ehu.es/) are shown in Table 4. Using the SopD outer primers (F3 and B3), positive amplification (indicated by the plus sign) was observed for all *Salmonella enterica* subsp. *enterica* serovars (Table 4). As expected, the designed primers showed no cross-reactivity with other bacterial genomes (indicated by the minus sign, Table 4).

**Figure 8:**
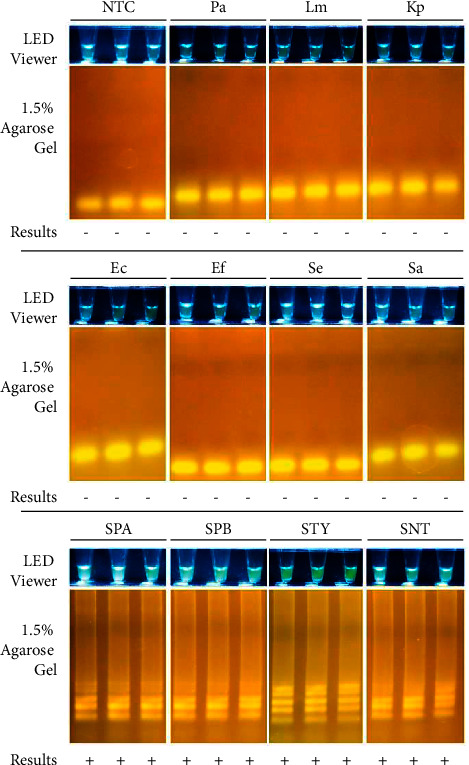
Assay specificity. Sample (2.5 *μ*L) was added to 10 *μ*L reagent mix then incubated at 65°C for 60 minutes, 80°C for 2 minutes, and cooled to 4°C for 10 minutes using T100 Thermal Cycler (S/N 621BR14432; Bio-Rad Laboratories, Inc., CA, USA). Results were viewed using the EasyViewTM LED Transilluminator (Manila HealthTek, Inc., Marikina, Philippines) which has a blue LED light and yellow plastic gel filter, and through agarose gel electrophoresis (1.5% agarose, 135 V for 25 minutes). *Salmonella enterica* subsp. *enterica* positives appear bright yellow green. Positive control panel includes the following: SPA-*Salmonella enterica* sv. Paratyphi A (typhoidal), SPB-*Salmonella enterica* sv. Paratyphi B (typhoidal), STY-*Salmonella enterica* sv. Typhi (typhoidal), and SNT-*Salmonella enterica* sv. Typhimurium (nontyphoidal). Negative control panel includes the following: Pa–*Pseudomonas aeruginosa*, Lm–*Listeria monocytogenes*, Kp–*Klebsiella pneumoniae*, Ec–*Escherichia coli*, Ef–*Enterococcus faecalis*, Se–*Staphylococcus epidermidi*s (Gram-positive bacteria), and Sa–*Staphylococcus aureus*. Samples were assayed in triplicate. All images were taken using a Canon EOS 750D camera. Interpretation of results are as follows: (+)–Positive using the LED transilluminator and positive in 1.5% AGE; (−)–Negative using the LED transilluminator and negative in 1.5% AGE.

The results of *in vitro* specificity testing showed that the assay could specifically amplify *Salmonella enterica* subsp. *enterica* serovars Typhi, Paratyphi A, Paratyphi B, and Typhimurium (Figure 8). No amplification was observed in the negative control panel samples. Hence, there was an agreement between the *in silico* and *in vitro* tests using the available control panels.

**Figure 9:**
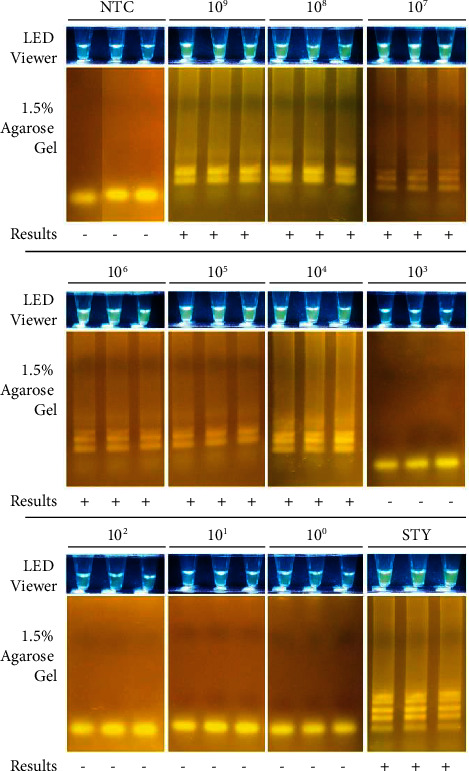
Assay sensitivity. Sample (2.5 *μ*L) was added to 10 *µ*L reagent mix then incubated at 65°C for 60 minutes, 80°C for 2 minutes, and cooled to 4°C for 10 minutes using T100 Thermal Cycler (S/N 621BR14432; Bio-Rad Laboratories, Inc., CA, USA). Samples used were the No Template Control (NTC), *Salmonella enterica* subsp. *enterica* sv. Typhi DNA (STY), and Positive Control (PC). The following concentrations of PC were used in triplicate: 10^9^, 10^8^, 10^7^, 10^6^, 10^5^, 10^4^, 10^3^, 10^2^, 10^1^, and 10^0^ copies per *μ*L. Results were viewed using the EasyView™ LED Transilluminator (Manila HealthTek, Inc., Marikina, Philippines) which has a blue LED light and a yellow plastic gel filter, and through agarose gel electrophoresis (1.5% agarose, 135 V for 25 minutes). *Salmonella enterica* subsp. *enterica* positives appear bright yellow-green. All images were taken using a Canon EOS 750D camera. Interpretation of results are as follows: (+)–Positive using the LED transilluminator and positive in 1.5% AGE; (−)–Negative using the LED transilluminator and negative in 1.5% AGE.

In this study, the analytical sensitivity was determined based on three replicates of nine sample concentrations (copies per *μ*L, cp/*μ*L) of the positive control. The *Salmonella* QUASR LAMP assay was able to detect down to 10^4^ cp/*μ*L of DNA target, in three out of three replicates (Figure 9).

*Salmonella* spp. can be detected in humans by testing clinical specimens such as blood and stool. Since PCR and QUASR LAMP are both molecular assays, the required volume for testing is minimal (200–500 *μ*L for blood and >1 g for stool). A study using real-time PCR amplification for the detection of invasive *Salmonella* serovars showed better limit of detection (LOD) values [26]. The number of copies of target DNA detected for *S.* Paratyphi A was about thirty-nine copies per mL of blood. The *S.* Typhi positive amplifications showed a LOD of about sixty copies per mL of whole blood. The analytical sensitivity of the developed *Salmonella* QUASR LAMP assay can be further improved to be up to par with rt-PCR values. Through the inclusion of detergent additives such as Triton X-100 and dimethyl sulfoxide (DMSO) in the reagent mixture, the analytical sensitivity of the assay can be enhanced [27].

## 4. Conclusions and Recommendations

Quenching of unincorporated amplification signal reporters in loop-mediated isothermal amplification (QUASR LAMP) has been demonstrated to be useful in detecting *Salmonella enterica* subsp. *enterica* DNA. This study verified that QUASR LAMP provides a closed-tube, target-specific endpoint for visual detection. It has discrimination between positive and negative samples and requires minimal instrumentation.

The developing *Salmonella* QUASR LAMP assay has applications in testing food, environmental, and clinical samples for the presence of *Salmonella enterica* subsp. *enterica*. Rapid, sensitive, and specific detection of *Salmonella enterica* infections may lead to proper clinical management and better outcomes. Furthermore, the QUASR LAMP assay takes only about 60 minutes for a test, while PCR assays usually take 80 to 90 minutes, and culture techniques can take days before getting conclusive results. Thus, the developed assay can be used in low-resource settings as an alternative to PCR and traditional microbiological techniques.

Additional studies are being done to improve the current limit of detection of the assay (10^4^ cp/*μ*L) through the addition of detergents like DMSO and Triton X-100. QUASR-LAMP detection can also be improved by using an algorithm that utilizes chromaticity to analyze the fluorescence signal (e.g., phone or web applications). Analytical specificity data can also be strengthened by testing a wider panel of positive controls (i.e., other *Salmonella enterica* subsp. *enterica* serovars) and negative controls (i.e., other closely related organisms). The assay can also be further developed and optimized to perform multiplex detection of different *Salmonella enterica* subsp. *enterica* serovars or other closely related enteric bacteria and viruses. QUASR LAMP, coupled with a direct rapid method for nucleic acid isolation, can further improve the assay setup by minimizing the complexity of sample preparation and reducing the time of analysis. The applicability of the developed assay can be determined by testing clinical specimens such as blood and stool, as well as by testing food and environmental samples.

## Figures and Tables

**Table 1:** Nucleic acid sequences of the generated SopD LAMP primer set. Four types of LAMP primers were designed using Primer Explorer v.5 (Fujitsu Ltd, 2015) based on the following six distinct regions of the target gene: the F3c, F2c, and F1c regions at the 3ʹ side and the B1, B2, and B3 regions at the 5′ side.

Label	Tm (°C)	GC content (%)	Length (bp)	5′ G	3′ G	Sequence (5′ ⟶ 3′)
SopD_S1_F3	59.3	61	18	−5.04	−6.85	CGAAGATGACCTGGCACC
Sop_DS1_B3	59.1	55	20	−5.18	−5.69	GCGACTTCACCTTGTACCTC
SopD_S1_FI						CCTCTGCGGGACGCATCATC
P (F1c + F2)						AAGTGACGGGAAATGCACAA
F1c	64.8	65	20	−5.19	−3.8	CCTCTGCGGGACGCATCATC
F2	60.0	45	20	−4.41	−5.57	AAGTGACGGGAAATGCACAA
SopD_S1_BI						GCTTGTCGAATGGCAGGACTCA
P						—
(B1c + B2)						CGTAGGCTCAAAGTTACCGG
B1c	64.8	55	22	−5.4	−4.76	GCTTGTCGAATGGCAGGACTCA
B2	59.8	55	20	−5.35	−5.86	CGTAGGCTCAAAGTTACCGG

Primer sets were evaluated using the Oligo Calculator v. 3.27 (Kibbe WA, https://bpc.facilities.northwestern.edu/OligoCalc) such that hairpin formation and self-dimerization are minimized [22] and were ordered for synthesis from Eurogentec (Belgium).

**Table 2:** SopD QUASR LAMP fluorophore-labeled primer and quencher probe set. The 5′ end of the FIP primer was appended with the FAM reporter dye (a green fluorophore). A short quencher probe complementary to the 5′ end of the labeled primer (FIPc) was manually designed. The quencher probe was modified at the 3′ end with a dark quencher (e.g., Black Hole quencher (BHQ)).

Label	Tm (°C)	GC content (%)	Length (bp)	Sequence (5′ ⟶ 3′)
SopD_S1_FIPc				6FAM/ACCTGCAGCTCATTCTGAGCAG-TCAAAAACAACGGCTCCGG
SopD_S1_FIPc_Q	36	50	12	TCCCGCAGAGG/BHQ1

**Table 3:** *In vitro* specificity of the negative and positive control panels. Specificity testing included various available samples and strains containing the target sequence (positive control panel) as well as samples containing nucleic acids that were expected to present negative results in the assay (negative control panel).

Negative control panel				

Organism	Sample code	Accession number	Source	Characteristics
*Escherichia coli*	Ec	BIOTECH 1634	PNCM	Gram-negative bacterium, grown in nutrient agar
*Staphylococcus aureus*	Sa	BIOTECH 1582	PNCM	Gram-positive bacterium, grown in nutrient agar
*Staphylococcus epidermidis*	Se	BIOTECH 10098	PNCM	Gram-positive bacterium, grown in nutrient agar
*Pseudomonas aeruginosa*	Pa	BIOTECH 1335	PNCM	Gram-negative bacterium, grown in nutrient agar
*Klebsiella pneumoniae*	Kp	BIOTECH 1754	PNCM	Gram-negative bacterium, grown in nutrient agar
*Enterococcus faecalis*	Ef	BIOTECH 10348	PNCM	Gram-positive bacterium, grown in de man, rogosa and sharpe agar
*Listeria monocytogenes*	Lm	BIOTECH 1958	PNCM	Gram-positive bacterium, grown in nutrient agar
Positive control panel (*Salmonella enterica* subsp. *enterica* serovars)				
Organism	Sample code	Accession number	Source	Characteristics
*Salmonella enterica* sv. Typhimurium	SNT	ATCC 13311	Microbiologics, inc., MN, USA	Nontyphoidal
*Salmonella enterica* sv. Paratyphi A	SPA	ATCC 9150	NSRI	Typhoidal
*Salmonella enterica* sv. Paratyphi B	SPB	ATCC 8759	NSRI	Typhoidal
*Salmonella enterica* sv. Typhi	STY	BIOTECH 1768	NSRI	Typhoidal

PNCM - Philippine National Collection of Microorganisms, National Institute of Molecular Biology and Biotechnology, Laguna, Philippines. NSRI - Natural Science Research Institute, University of the Philippines Diliman, Quezon City, Philippines.

**Table 4:** *In silico* PCR amplification of SopD F3 and B3 primers. *In silico* PCR amplification was done against different bacterial genomes using a freely available molecular biology resource for experiments (http://insilico.ehu.es/). Interpretation of results are as follows: (+) – Amplification was observed, (−) – No amplification was observed.

Organism	SopD F3 and B3 amplification (+/−)
*Escherichia coli*	−
*Klebsiella pneumoniae*	−
*Staphylococcus epidermidis*	−
*Staphylococcus aureus*	−
*Bacillus cereus*	−
*Enterococcus faecalis*	−
*Enterococcus faecium*	−
*Listeria monocytogenes*	−
*Streptococcus agalactiae*	−
*Streptococcus pneumoniae*	−
*Streptococcus pyogenes*	−
*Haemophilus influenzae*	−
*Pseudomonas aeruginosa*	−
*Acinetobacter baumannii*	−
*Neisseria meningitidis*	−
*Bacteroides fragilis*	−
*Bacillus anthracis*	−
*Yersinia pestis*	−
*Francisella tularensis*	−
*Salmonella bongori*	−
*Salmonella enterica* subsp. *arizonae* serovar 62: z4, z23:--	−
*Salmonella enterica* subsp. *enterica* serovar Cubana	+
*Salmonella enterica* subsp. *enterica* serovar Agona	+
*Salmonella enterica* subsp. *enterica* serovar Bareilly	+
*Salmonella enterica* subsp. *enterica* serovar Bovismorbificans	+
*Salmonella enterica* subsp. *enterica* serovar Choleraesuis	+
*Salmonella enterica* subsp. *enterica* serovar Dublin	+
*Salmonella enterica* subsp. *enterica* serovar Enteritidis	+
*Salmonella enterica* subsp. *enterica* serovar Gallinarum	+
*Salmonella enterica* subsp. *enterica* serovar Gallinarum/pullorum	+
*Salmonella enterica* subsp. *enterica* serovar Heidelberg	+
*Salmonella enterica* subsp. *enterica* serovar Javiana	+
*Salmonella enterica* subsp. *enterica* serovar Newport	+
*Salmonella enterica* subsp. *enterica* serovar Paratyphi A	+
*Salmonella enterica* subsp. *enterica* serovar Paratyphi B	+
*Salmonella enterica* subsp. *enterica* serovar Paratyphi C	+
*Salmonella enterica* subsp. *enterica* serovar Pullorum	+
*Salmonella enterica* subsp. *enterica* serovar Schwarzengrund	+
*Salmonella enterica* subsp. *enterica* serovar Thompson	+
*Salmonella enterica* subsp. *enterica* serovar Typhi	+
*Salmonella enterica* subsp. *enterica* serovar Typhimurium	+

## Data Availability

The data used to support the findings of this study are included within the article.
